# Sustainable Approach for Improving Tool Life and Surface Quality During Diamond Cutting of Ultra-Low-Expansion Glass Using Laser Assistance

**DOI:** 10.3390/mi17050633

**Published:** 2026-05-21

**Authors:** Han Zhang, Shizhen Zhu, Xiao Chen, Chuangting Lin

**Affiliations:** 1 School of Material Science and Engineering, Beijing Institute of Technology, Beijing 100081, China; zhanghan@feilihua.com (H.Z.); zhusz@bit.edu.cn (S.Z.); 2 Hubei Key Laboratory of Advanced Optical Quartz Materials Technology, Hubei Feilihua Quartz Glass Co., Ltd., Jingzhou 434000, China; 3 Innovation Center for Special Optical Glass Materials Technology, Jingzhou 434000, China; 4 Key Laboratory of Modern Manufacturing Quality Engineering in Hubei Province, Hubei University of Technology, Wuhan 430068, China; chenxiao1987jz@163.com; 5 College of Marine Equipment and Mechanical Engineering, Jimei University, Xiamen 361021, China

**Keywords:** sustainability, tool life, surface quality, in situ laser assisted diamond cutting, ultra-low-expansion glass

## Abstract

Ultra-low-expansion (ULE) glass serves as a critical material in high-precision optical devices and semiconductor manufacturing; however, its inherent hardness and brittleness pose significant challenges for machining processes. During the diamond cutting of ULE glass, severe tool wear emerges as the primary factor limiting machined quality, which not only shortens tool life but also prolongs subsequent polishing time, thereby increasing processing costs and hindering sustainable manufacturing. To address this challenge, in situ laser assisted diamond cutting (LADC) has emerged as a promising technique for the sustainable machining of difficult-to-machine materials. In this study, for achieving sustainable machining of ULE glass, the effects of cutting speed on surface roughness and tool wear were systematically investigated. To determine the optimal parameter combination for minimizing surface roughness and tool wear simultaneously, an integrated optimization approach combining artificial neural network (ANN) and non-dominated sorting genetic algorithm II (NSGA-II) was employed. The experimental results indicated that a spindle speed of 2900 rpm and a feed speed of 1.1 mm/min was ascertained as the optimum combination to attain the desired outcomes for in situ LADC of ULE glass. Under the optimum machining parameters, in situ LADC resulted in a 70.08% reduction in surface roughness and 61.24% reduction in tool wear compared to conventional diamond cutting (CDC). This study demonstrates that in situ LADC can be recognized as a promising sustainable machining technique for machining of ULE glass.

## 1. Introduction

ULE glass is a material with a wide range of applications, including optical devices and precision instruments. Due to its exceptional thermal stability and low thermal expansion coefficient, ULE glass plays a critical role in semiconductor manufacturing and optical metrology. For instance, ULE glass is the primary material for EUV photomasks, where its highly uniform thermal expansion coefficient ensures the fidelity of lithographic patterns even under extreme temperature variations [1,2,3]. As demand for more accurate and stable optical components increases, especially in the fabrication of EUV photomasks, achieving high precision in ULE glass machining becomes increasingly important.

Diamond cutting and then polishing is an efficient way to process ULE glass lenses. Diamond cutting is an efficient method for complex shape lenes, which has achieved the ultra-precision machining of various kinds of materials, such as aluminum and copper [4,5,6]. However, the surface roughness of optical glass exhibits a rapid increase beyond a cutting distance of 150 m due to accelerated tool wear [7]. It poses a formidable challenge for diamond cutting of hard and brittle materials (HBMs) due to severe tool wear, which degrades the surface quality [8,9,10]. Serious tool wear reduces tool life and poor machined surface quality prolongs polishing time, which increase the processing cost. Furthermore, as industries move toward sustainability, sustainable manufacturing is more and more important, which aims to create products to minimize costs while reducing the environmental impact [11,12]. Therefore, achieving high surface quality while reducing tool wear is important during diamond cutting.

In situ LADC is a promising manufacturing technology for HBMs that employs a focused laser beam passing through the diamond tool to heat and soften materials, resulting in improved surface quality and extended tool life [13,14,15]. Laser assistance prevents subsurface crystal bending and minimizes subsurface damage effectively during diamond cutting of binderless tungsten carbide, and a surface finish of 0.97 nm in Sa was achieved by utilizing optimal laser power [16,17,18]. Sim et al. [19] established a ductile machining process of piezoelectric single crystal based on the method of LADC, achieving high-quality machining of various shapes, such as double-sided and multi-scale microstructures. Luo et al. [20] investigated the machinability enhancement of ZnS during in situ LADC. Experimental results revealed that with laser assistance, critical cutting increased by 57.99% and surface quality improved by 73.58% compared to CDC. In addition to improving the machined surface quality, the in situ LADC technology has shown great advantages in reducing tool wear. The tool wear conditions during in situ LADC of high-entropy alloy CoCrFeMnNi were compared with CDC. Experimental results revealed that with a 35% improvement in surface quality, tool wear decreases by above 35% during in situ LADC [21]. Huang et al. [22] adopted in situ LADC for ultra-precision machining nitriding mold steel. Experimental results show that the machined surface roughness Ra is decreased from 29 nm to 12 nm compared with ordinary cutting, and the tool life increased significantly by 29%. By incorporating laser assistance, the plastic deformability of fused silica is enhanced, which gives rise to an increase in critical cutting depth from 82.06 nm to 324.03 nm, and surface roughness in Sa of 13.8 nm was successfully attained [23,24].

The surface quality and tool life during diamond cutting of HBMs are significantly influenced by various parameters [25,26]. A high feed speed and a high rotation speed within a certain range minimize tool wear during diamond cutting of large silicon optics due to the reduction in machining time [27]. During in situ LADC, both feed speed and spindle speed affect the cutting distance and the temperature in the cutting zone significantly, resulting in different surface quality and tool wear [28,29]. Optimization of cutting parameters is crucial for improving machining sustainability. However, the influence of cutting conditions on tool life and surface quality during in situ LADC of ULE glass has received limited research attention.

In order to study the sustainability of in situ LADC in high-precision machining of ULE glass, a novel integrated framework specifically tailored to ULE glass is established. The influence of cutting speed on surface quality and tool wear is investigated through systematic experiments, utilizing a hybrid approach that combines ANN and NSGA-II. This methodology is further distinguished by the incorporation of LIME (Local Interpretable Model-Agnostic Explanations) to provide physical insights into the influence of laser assistance. The method, experimental procedure, results and discussion regarding this physics-informed data-driven strategy, and conclusions are given in detail in the following sections.

## 2. Method

ANN models were developed as surrogate models to predict both surface roughness and tool wear. Through the application of Bayesian optimization technology and the cross-validation method, various ANN structures were examined to identify the optimal hyperparameters that yielded the highest performance. Mean square error (MSE) was utilized as a quantitative measure to construct highly efficient ANN models [30]:

(1) $$MSE=\frac{1}{N}\sum_{i=1}^{N}\{y_{i}-{\hat{y}}_{i}{\}}^{2}$$

where $y_{i}$ and ${\hat{y}}_{i}$ are actual and predicted values, respectively. N is the total number of observations.

However, it fails to demonstrate the realism of local feature explanations due to the lack of the analysis of feature importance of the ANN model, even if the model exhibits sufficient generalization capability on the test dataset. Hence, it becomes crucial to utilize LIME for post hoc explanations [31]. LIME leverages a well-trained ANN model to train a simple local surrogate model, specifically for explaining individual samples, as depicted in Equation (2). This approach facilitates more comprehensive feature analysis and offers additional explanations for subsequent optimization outcomes.

(2) $$\xi \left(x\right)=argmin_{g\in G}L\left(f,g,\pi_{x}\right)+\Omega \left(g\right)$$

where *L* represents a designated loss function indicating the degree of dissimilarity between the surrogate model *g* and the original model *f*. *g* is the selected surrogate model that approximates the behavior of *f* within the immediate vicinity of *x*. $\pi_{x}$ refers to the proximity measure, which determines the significance of the generated samples within the local neighborhood. Ω(*g*) corresponds to a complexity term of the model, which is expected to be low to provide a more comprehensible explanation.

**Figure 1:**
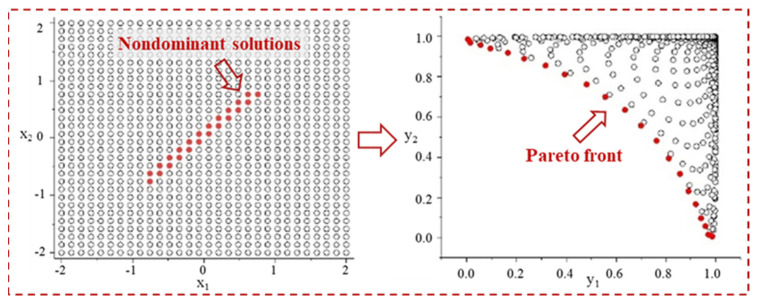
Illustration of Pareto front.

**Figure 2:**
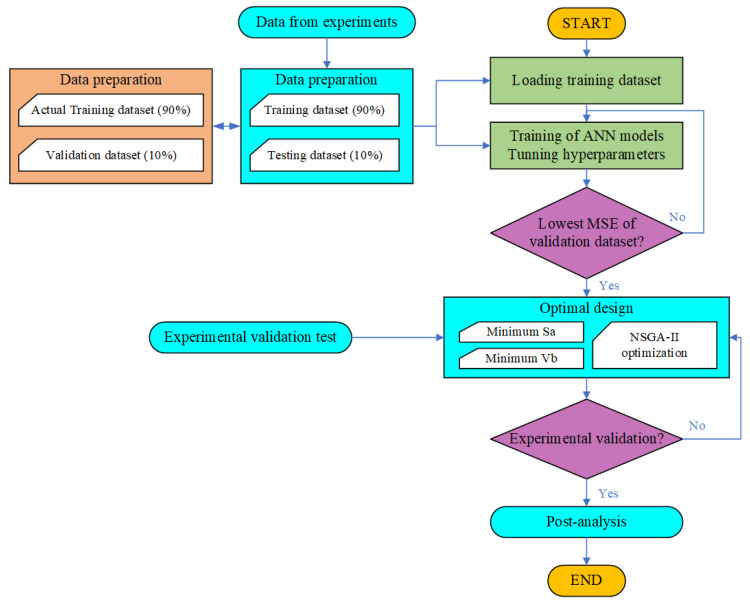
Flowchart of the multi-objective optimization.

The NSGA-II was employed to attain a trade-off between the cost of the finished product and its classical benefits [32]. To achieve the balance between tool wear and surface quality, the studied ANN models were integrated into the NSGA-II optimization method as the objective function to be minimized. The set of optimal solutions, which considers conflicts and incompatibilities between different targets, is referred to as the Pareto front, as depicted in Figure 1. The flowchart in Figure 2 outlines the optimization methodology employed. The Pareto front was utilized to identify optimal solutions that strike a balance between surface roughness and tool wear, all within the specified range constraints. To validate the effectiveness of the numerical model and the optimal values of surface roughness and tool wear, a verification experiment was conducted.

## 3. Experimental Procedure

**Figure 3:**
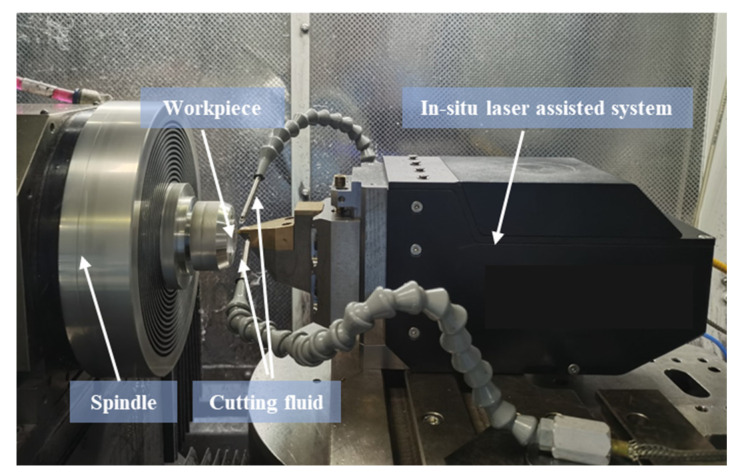
Setup for diamond cutting experiments.

A laser-assisted machining test was set up as shown in Figure 3, where an in situ laser-assisted system was integrated into an ultra-precision machine tool (Precitech Nanoform X, Keene, NH, USA). A ULE glass plate (Hubei Feilihua Quartz Glass Co., Ltd., Jingzhou, China) with a diameter of 25.4 mm and a thickness of 4 mm was employed as the workpiece. A single crystalline diamond tool with a nose radius of 1.5 mm, a rake angle of −65°, and a flank angle of 10° was used as the cutting tool. The selection of these specific tool geometries was based on our previous experimental validations, which confirmed that this configuration is particularly conducive to inducing plastic deformation in hard and brittle materials like ULE glass [24]. A laser beam was directed through a diamond tool with a focus diameter of 80 μm to heat the workpiece.

The cutting speed has a substantial impact on the determination of the cutting distance, directly affecting tool life and surface quality. Therefore, the spindle speed and feed speed were selected as the key machining parameters for investigation. To ensure an efficient experimental design, the Taguchi method was employed to plan and execute the experiments. An $L_{9}(2^{3})$ standard orthogonal array was constructed. The selection of the fixed parameters was based on preliminary experiments and established empirical data from relevant studies [33,34]. As shown in Table 1, the cutting depth was fixed at 2 μm, while the laser power was set to 10 W, and the cutting distance was maintained at 2.0 km. This combination of parameters was verified to enable stable ductile-regime cutting of ULE glass, effectively avoiding brittle fracture and ensuring the integrity of the machined surface. Analysis of Variance (ANOVA) was employed to evaluate the impact of each factor on surface quality and tool wear. The Signal-to-Noise (S/N) ratio was utilized to assess the influence of cutting conditions on the characteristic indexes, following the smaller-the-better model.

The cross-section profile of the machined surface roughness was measured by the white light interferometer (ZYGO Newview 9000, Middlefield, CT, USA). The micromorphology of the worn tool after diamond cutting was identified by the optical microscope (ZEISS Axiolab 5, Oberkochen, Germany).

## 4. Results and Discussion

### 4.1. Effect of Cutting Condition on Tool Wear and Surface Quality

Table 2 presents the results obtained from nine in situ LADC experimental trials. ANOVA analysis and the S/N ratio were employed to examine the effects of spindle speed and feed speed on tool wear and surface quality. The results from Table 2 are further analyzed in Table 3, which includes information such as degrees of freedom, sum of squares, sum of mean squares, and contribution. The analysis reveals that spindle speed contributes to 31.18% of the tool wear, while feed speed contributes to 58.29%. This indicates that both spindle speed and feed speed have a significant impact on tool wear, with feed speed having a slightly higher contribution. This is because the spindle speed determines the duration of the machining process, while the feed speed affects the material removal rate. Both factors contribute to tool life. Regarding surface roughness, the contribution percentages are found to be 65.95% for spindle speed and 3.36% for feed speed. Consequently, spindle speed has a greater influence on surface quality compared to feed speed.

**Figure 4:**
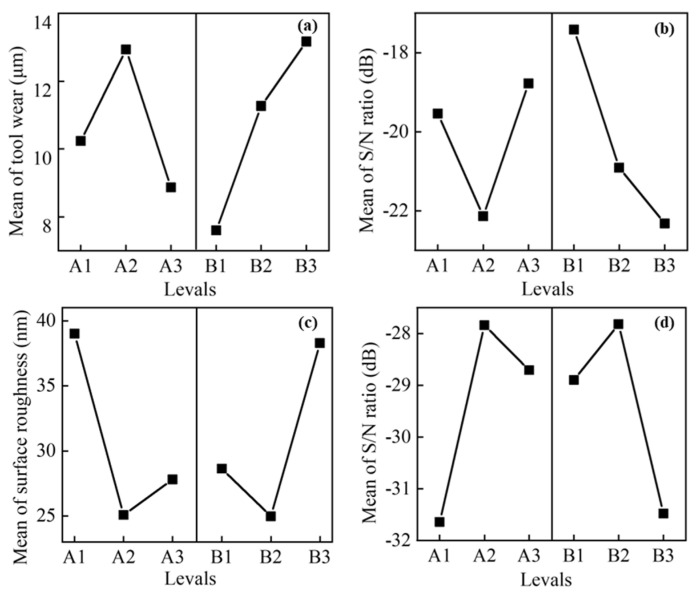
Main effect plots. (**a**) The effect of input factors on tool wear. (**b**) The mean S/N ratios corresponding to tool wear. (**c**) The effect of input factors on surface roughness. (**d**) The mean S/N ratios corresponding to surface roughness.

Table 4 presents the calculated average S/N ratio, tool wear, and surface roughness for different levels of each factor. The impact of cutting conditions on tool wear and surface quality was assessed using the main effect plot, as depicted in Figure 4. The analysis of the main effect plot reveals that tool wear is minimized at the third level of spindle speed (A) and the first level of feed speed (B). This can be attributed to the fact that the higher spindle speed reduces the machining time at the same cutting distance, thereby reducing the accumulation of heat around the cutting region. Consequently, the generation of dynamic hard particles decreases, thereby improving the longevity of the diamond tool. In a previous study, a high feed speed was found to minimize tool wear due to a decrease in track length [35]. However, the cutting distance was kept constant across different experiments in the current study. Hence, an increase in feed speed leads to larger tool wear due to the larger feed rate, resulting in an increased material removal volume. On the other hand, surface roughness is found to be minimized at the second level of spindle speed (A) and the second level of feed speed (B). The increase in spindle speed contributes to a decrease in the maximum undeformed chip thickness, thereby reducing surface roughness to some extent. However, it should be noted that excessively high spindle speed can compromise the stability of the cutting process, thereby negatively impacting machining quality. The higher feed speed is found to reduce diamond tool wear when considering the same cutting distance, thereby enhancing surface quality. However, as the feed speed increases, the surface finish tends to deteriorate due to the increase in the maximum undeformed chip thickness. This suggests that there is a trade-off between feed speed and surface roughness, where higher feed speeds can improve tool life but may result in a decline in surface finish due to the increased chip thickness.

According to Figure 4, the optimal parametric combination for minimizing tool wear is identified as A3B1, corresponding to a spindle speed of 3000 rpm and a feed speed of 1 mm/min. Conversely, the optimal parametric combination for minimizing surface roughness is found to be A2B2, with a spindle speed of 2000 rpm and a feed speed of 2 mm/min. It is important to note that the conditions that result in the minimum tool wear and the minimum surface roughness are not the same. This highlights the need to identify optimal conditions that can simultaneously achieve all desired goals through multi-objective optimization.

### 4.2. Model Fitting and Optimization

The ANN model is characterized by a range of sensitive hyperparameters, such as the number of epochs, hidden layers, hidden units, and choice of activation function [36], as illustrated in Table 5. The process of optimizing these hyperparameters helps identify an optimal combination that maximizes the model’s performance and generalization capability. This was accomplished through the effective utilization of Bayesian optimization technology and cross-validation methods with the principal error metric.

**Figure 5:**
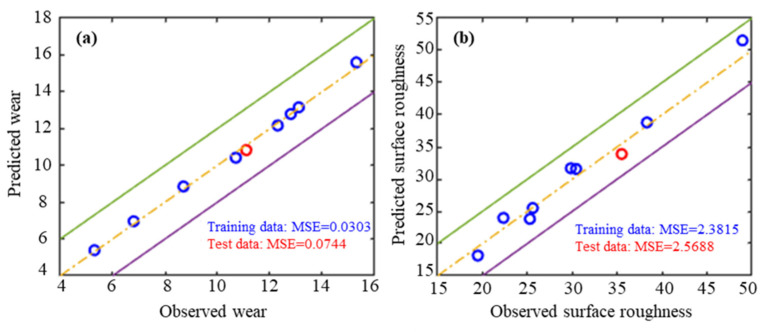
ANN model performance. (**a**) Tool wear; (**b**) surface roughness.

Figure 5 displays the performance of the ANN models in predicting tool wear and surface roughness, as assessed using both training and testing datasets. In terms of tool wear, the ANN model yields MSE values of 0.0303 on the training dataset and 0.0744 on the test dataset. For surface roughness, the ANN model achieves MSE values of 2.3815 and 2.5688 for the training and testing datasets, respectively. These results indicate a strong correlation between the numerical predictions and the actual values.

**Figure 6:**
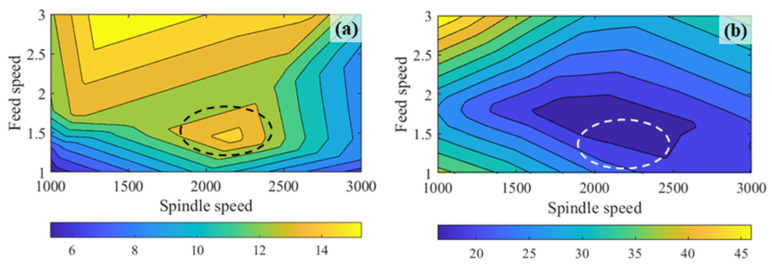
Distribution of prediction. (**a**) Tool wear; (**b**) surface roughness.

Figure 6 displays the distribution of tool wear and surface roughness, providing a comprehensive visualization of the impact of feed speed and spindle speed. Notably, Figure 6 reveals that the changes in tool wear and surface roughness do not consistently align. Within the highlighted circular region, a decrease in tool wear does not correspond to a reduction in surface roughness. Consequently, achieving a harmonious equilibrium between these two indicators became the focal point of this research.

**Figure 7:**
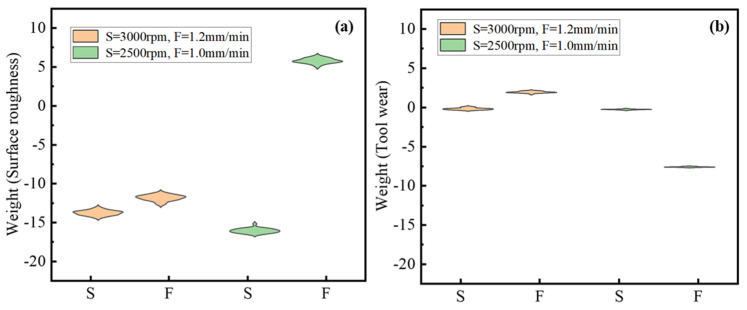
Feature importance. (**a**) Surface roughness; (**b**) tool wear.

Since LIME introduces random perturbations to the selected samples during each iteration, the surrogate model generated is not consistent across iterations. Utilizing the LIME approach, 30 local interpretations were conducted for two positions, namely S = 2500 rpm, F = 1.0 mm/min, and S = 3000 rpm, F = 1.2 mm/min. The resulting distribution of weights is illustrated in Figure 7. As depicted in Figure 7a, increasing the spindle speed appropriately proves advantageous in reducing surface roughness. Furthermore, it is worth noting that the spindle speed exerts a greater influence compared to the feed speed. When S = 2500 rpm, the impact of the feed speed shifts in a positive direction, indicating that an increase in feed speed leads to an elevation in surface roughness. Concerning the impact of these two features on tool wear, their weights are smaller compared to their influence on surface roughness, as demonstrated in Figure 7b. This discrepancy may arise due to the absence of normalization in the calculation of influence for both surface roughness and tool wear. Consequently, the numerical values of tool wear are relatively smaller, resulting in correspondingly reduced influence weights. When S = 2500 rpm and F = 1.0 mm/min, the effect of feed speed on tool wear and surface roughness exhibits opposite trends. As mentioned before, it is apparent from the LIME feature importance analysis that the impact of the same feature on surface roughness and tool wear displays contrasting patterns when assuming similar values. This finding further confirms the necessity of subsequent multi-objective optimization.

Therefore, the multi-objective problem is formulated as follows, where $\hat{Vb}$ and $\hat{Sa}$ are models predicting the predicted values of surface roughness and tool wear, respectively. The problem constraints are expressed as follows:

(3) $$\begin{array}{c} Objectives:\\ Minimize\;\hat{Vb}\\ Minimize\;\hat{Sa} \end{array}$$

(4) $$\begin{array}{c} subject\;to\;constraints:\\ 1\leq F\leq 3\\ 1000\leq S\leq 3000 \end{array}$$

**Figure 8:**
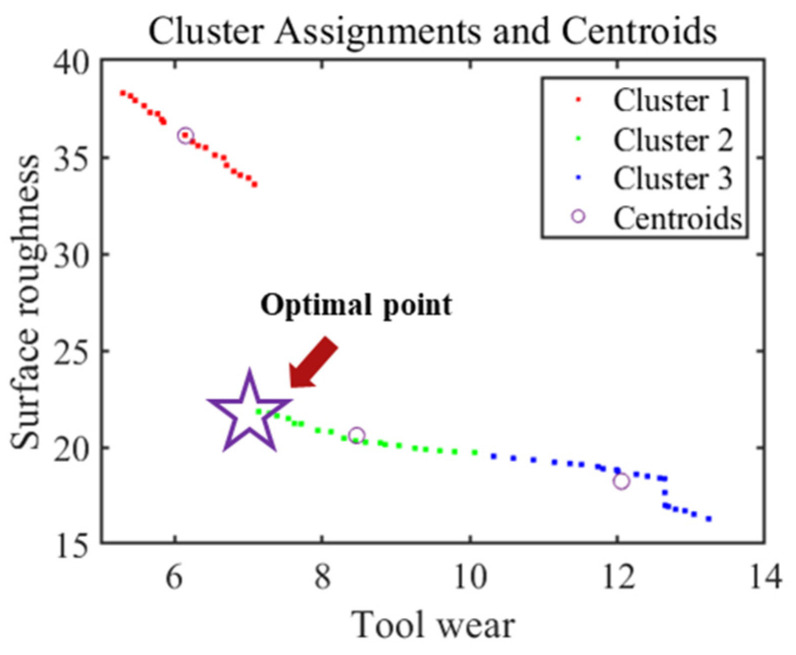
Pareto front of non-dominated results.

In this study, favorable convergence was achieved by employing a population size of 60, a maximum generator limit of 100, a crossover rate of 0.8, a mutation rate of 0.2, and a selection rate of 0.2. Figure 8 showcases the relationship between predicted surface roughness and tool wear on the Pareto front and the corresponding dominant solutions. Utilizing the K-means method, the Pareto front can be categorized into three distinct clusters, each exhibiting varying levels of objective values. Moreover, Figure 8 reveals a clear demarcation between cluster 1 and cluster 2. Within this boundary, there is a noticeable decrease in surface roughness without a significant increase in tool wear. Consequently, the boundary point denoted by a star between cluster 1 and cluster 2 is considered the optimal solution for this study.

**Figure 9:**
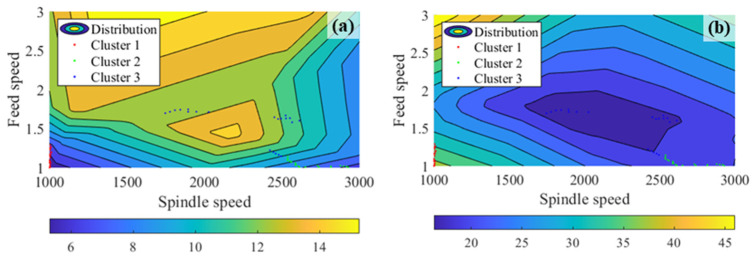
Distribution of prediction with cluster information. (**a**) Tool wear; (**b**) surface roughness.

By incorporating cluster information into the analysis of non-dominant solutions, it is observed that the input variable values associated with each cluster exhibit remarkable similarities, as depicted in Figure 9. Notably, it becomes evident that a relatively well-balanced trade-off between surface roughness and tool wear is attained in cluster 2 when the feed speed falls within the range of 1.0 to 1.2 mm/min and the spindle speed lies between 2500 and 3000 rpm.

To validate the Pareto optimal solutions, a confirmatory experiment was conducted, employing a spindle speed of 2900 rpm and a feed speed of 1.1 mm/min according to Figure 9. The predicted mean values of surface roughness and tool wear obtained through NSGA-II optimization are 20.15 nm and 7.14 μm, respectively. To assess the accuracy of the model, three confirmatory experiments were conducted with the optimal combination of parameters at a cutting distance of 2.0 km. The experimental results yield an average surface roughness of 18.63 nm and tool wear of 6.83 μm, which is closely aligned with the predicted values. The errors for surface roughness and tool wear were calculated to be 8.16% and 4.54%, respectively. Hence, the synergistic employment of NSGA-II and ANN holds promise for effectively attaining the desired surface roughness and tool wear during in situ LADC. Furthermore, to further verify the superiority of the in situ LADC technology in the sustainable machining of ULE glass, a CDC experiment under the same condition was carried out. The surface roughness obtained without laser assistance reached 62.27 nm, and the flank wear length of the diamond tool was 17.62 μm. Compared to CDC, in situ LADC resulted in a 70.08% reduction in surface roughness and 61.24% reduction in tool wear. Therefore, in situ LADC can be recognized as a promising sustainable machining technique for machining of ULE glass.

## 5. Conclusions

A novel integrated framework combining ANN, NSGA-II, and LIME was successfully implemented for the first time in the context of ULE glass machining. The impact of cutting speed on tool wear and surface roughness during in situ LADC was systematically examined, validating the effectiveness of this physics-informed data-driven approach in identifying the most favorable process parameters. The following findings can be summarized:

(1) ANOVA analysis reveals that both spindle speed and feed speed have a significant impact on tool wear and surface quality. Feed speed has a slightly higher contribution to tool wear, while spindle speed has a greater influence on surface quality compared to feed speed.
(2) The optimal parameter, as tool wear and surface roughness achieved their minimum values at the same time, is a spindle speed of 2900 rpm and a feed speed of 1.1 mm/min. The experimental results demonstrate that the synergistic employment of NSGA-II and ANN has an advantage in optimizing machining parameters and predicting minimum tool wear and surface roughness for in situ LADC of ULE glass.
(3) Under the optimum machining parameters, in situ LADC resulted in a 70.08% reduction in surface roughness and 61.24% reduction in tool wear compared to CDC. In situ LADC can be recognized as a promising sustainable machining technique for machining of ULE glass.

However, it should be noted that this study focuses on the optimization and validation of these performance improvements from a data-driven perspective. Consequently, a detailed investigation into the specific physical mechanisms governing tool wear is identified as a key focus of our future research.

## Figures and Tables

**Table 1:** Machining conditions for standard orthogonal array.

Machining Conditions	Parameters
Spindle speed (rpm)	1000/2000/3000
Feed speed (mm/min)	1.0/2.0/3.0
Cutting depth (μm)	2.0
Laser power (W)	10
Cutting distance (km)	2.0

**Table 2:** Machining conditions and experimental results.

No.	Machining Conditions		Experimental Results		
	Spindle Speed A (rpm)	Feed Speed B (mm/min)	Material Removal Volume ( ${mm}^{3}$)	Tool Wear Vb (μm)	Surface Roughness Sa (nm)
1	1 (1000)	1 (1.0)	4.05	5.3	38.3
2	1	2 (2.0)	8.11	12.3	29.8
3	1	3 (3.0)	12.16	13.1	48.9
4	2 (2000)	1	2.03	10.7	25.3
5	2	2	4.05	12.8	19.5
6	2	3	6.08	15.3	30.4
7	3 (3000)	1	1.32	6.8	22.3
8	3	2	2.63	8.7	25.6
9	3	3	3.95	11.1	35.5

**Table 3:** Response table of means and signal-to-noise ratios.

Factors	Level 1	Level 2	Level 3	Delta
	Tool wear			
Mean of S/N ratio (dB)				
A	−19.54	−22.14	−18.78	3.36
B	−17.42	−20.91	−22.32	5.07
Mean of tool wear (μm)				
A	10.233	12.933	8.867	4.067
B	7.600	11.267	13.167	5.567
	Surface roughness			
Mean of S/N ratio (dB)				
A	−31.64	−27.84	−28.71	3.80
B	−28.90	−27.82	−31.48	3.61
Mean of surface quality (nm)				
A	39.00	25.07	27.80	13.93
B	28.63	24.97	38.27	13.30

**Table 4:** ANOVA for tool wear and surface roughness.

Factor	D.O.E	Sum of Square	Sum of Mean Square	Contribution
Tool wear				
A	2	25.696	12.848	31.18%
B	2	48.042	24.021	58.29%
Error	4	8.678	2.169	10.53%
Total	8	82.416	39.038	100%
Surface roughness				
A	2	1983.1	991.56	65.95%
B	2	101.1	50.53	3.36%
Error	4	922.8	230.70	30.69%
Total	8	3007.0	1272.79	100%

**Table 5:** Hyperparameters for ANN.

Parameter Used	Grid Space	Vb Results	Sa Results
Epochs	Maximum 300	200	100
Number of layers	Maximum 3	2	2
Number of units	Maximum 30	[10, 10]	[10, 10]
Activation function	[‘ReLU’, ‘Tanh’, ‘Sigmoid’]	‘Tanh’	‘ReLU’

## Data Availability

The original contributions presented in this study are included in the article. Further inquiries can be directed at the corresponding author.

## Abbreviations

The following abbreviations are used in this manuscript:

ULE	Ultra-low-expansion
LADC	Laser-assisted diamond cutting
ANN	Artificial neural network
NSGA-II	Non-dominated sorting genetic algorithm II
CDC	Conventional diamond cutting
HBMs	Hard and brittle materials
MSE	Mean square error
LIME	Local Interpretable Model-Agnostic Explanations
ANOVA	Analysis of Variance
S/N	Signal-to-Noise
